# The Claims of the Medical Profession

**Published:** 1853-09

**Authors:** 


Art. V.—*The Claims of the Medical Profession.* The Annual Address Delivered before the New York State Medical Society and Members of the Legislature, at the Capitol, February, 1853, by A. Clark, M.D., President of the Society, and Professor of Physiology and Pathology in the College of Physicians and Surgeons of the State of New York, etc. Published by the Society and by the Legislature. Albany: Revised Edition, 1853. 8vo., pp. 24.

This is an address of great beauty and force. On every page it exhibits good taste, correct, vigorous thinking, and just sentiments, eloquently expressed. We wish it could be read by every physician in our country. Nay, we wish every body who expects ever to have need of the physician's skill, could read it. As it cannot be so widely circulated, we propose to give as much diffusion as we can to some of the excellent views contained in it.

Dr. Clark is speaking of the repeal of the laws which, in many of the States, once regulated the practice of medicine. He says —

In simple truth, medicine, on her own behalf, does not deplore the change. While led by the parental hand of government, it felt itself perhaps a child ; but rejected and spurned by its parent, of a sudden it discovered its manhood and its strength. It is for society alone we mourn. They, by such legislation, are left a prey to the pretender, who, by the boldest promises and most daring lies, can most impose on their credulity. But the profession is hardly reached by it ; that has a hold on the affections of man which is scarcely less firm than the love of life itself.

The feeling with which the physician is cherished is,then, too near akin to selfishness ever to die out, while men believe that skill is the legitimate child of knowledge and experience. Young transplanted trees are boxed in to protect them from injury, or tied to supports to brace them against the violence of the winds ; but when they have sent their roots deep, and their branches high, the trunk becomes strong, and they stand hardly less firm than the earth which nourishes them. So our profession, springing from a social necessity, protected by the hand of law till it has become deep rooted in the constitution of society, and has sent forth its branches to overshadow and bless it, though now the protection be withdrawn, yet will it stand unshaken till society itself is disorganized. We need then no protection—we ask for none. We ask for no other recompense than that which suffering humanity cheerfully pays to the hand that alleviates.

And yet the incongruities of legislation may merit a passing notice. Out of the many that offer themselves I will select but two. While government carefully defends the people against their own ignorance, by proving and stamping every penneyweight of gold and every pennyworth of copper that is put into circulation as money, and with earnest severity punishes every counterfeiter who cheats us of a single dollar, it refuses its sanction to those who offer ro judge for that same people of all that relates to the deep mysteries of life and disease ; it has no punishment, no reproof even, for the counterfeit physician that cheats us of our life. It inspects drugs and medicines, destroying all that are not of standard purity, and yet it cares nothing who uses them ; thus the government officer is made to sharpen the sword, and throw it into the circle, indifferent whether it fall into the hands of the trained and tried soldier, or into those of the highwayman.

Medicine, Dr. Clark shows, is making progress, notwithstanding the neglect of government, and all the quackery of pretenders. It is conferring vast benefits upon society, notwithstanding society in its collective capacity refuses to foster and protect it.

But I shall be told, doubtless, that I am claiming for my profession more than we have any fair right to; that society has improved in all its relations, and that to these improvements are due, in fair proportion, the results which I have quoted. Let us consider for a little in what these improvements consist. Within one hundred and fifty years the arts have reduced the cost of many of the necessaries of life; but then the necessaries of life have been actually multiplied by this process of reduction, and food, the first of necessaries has not been cheapened; if its relative money price has not materially changed, its labor cost is greater. The home condition of the laborer —I speak only of the countries from which I have drawn statistics—is more miserable than it was a century and a half ago. The rich have, it is true, become richer, but the poor have at the same time become poorer; in other words, wealth has greatly increased, but it is not distributed in other countries as it is in our own. Who that has visited the homes of labor in England and France, will believe that the over-crowded, half-clad, half-fed population of a manufacturing town can be compared in domestic comfort with the laboring classes of other times, when the housewife wrought out of the noisy wheel and loom the honest, warm, abundant homespun; when the labors of the field brought to a country, not overpopulated, abundance of food; when labor had not yet destroyed its compensation by rivalry with itself; when the infirm poor were not yet so numerous that the benevolent rich could not learn their condition and supply their wants. Who will believe that the crowded, hot, dusty, ill-ventilated manufactory can contribute to health like the open field, where men once labored, with its fresh breeze and its sunshine. The better and middle classes have always been long-lived. *Their* home condition may have been improved in the period referred to; but have they gained as much as the many, the laborers, have lost? I confidently believe that so far from their being a betterment in the social condition of Europe within a century and a half, when a fair balance is struck, it will be found that things personal contribute less than formerly to prolong life. Still, it cannot be denied, that in the general improvement of society, something has been done for this great object. It is in cities chiefly that these important changes are seen; and even there they are confined mostly to the rich, or at best are brought by the rich only to the doors of the poor, beyond which they rarely strive to pass. Staying as far as possible the spread of pestilence; improved ventilation in the widening of the streets, and in the better constructions of dwellings and public buildings; diminishing the causes of disease by the removal of filth, and by a judicious drainage; and the encouragement of personal cleanliness, by making water abundant and bathing cheap; these, no one will deny, are benefits, solid benefits. But *all that is valuable in them is based on principles elaborated and expounded by the medical profession.* Even the details of the plans by which the public have realized these benefits, have in many instances been prescribed by the profession. There is an applied recognition of this fact, in the name "medical police," which is given to the department that governs most of these things, and still more in the acknowledged necessity that their supervision be mainly intrusted to an "inspector" chosen from the medical profession. These are medical facts popularized, as are a thousand other medical facts in hygiene and the laws of regimen. May we not, then, freely imparting to the public, as we do, the advantages derivable from these things, may we not ask to be remembered as the authors of the doctrines from which thes benefits flow?

There is another view of this subject. Among the causes of *tubercular consumption* we hear enumerated imperfect protection either by house or clothing, against the vicissitudes of weather; scanty and innutritious food; imperfect ventilation; vitiated air; dwelling in dark, damp places; indifference to personal cleanliness. When it is remembered these are important points among the particulars in which it is claimed that the condition of society has been so greatly ameliorated, it would be expected that this formidable malady must gradually recede before the advancing improvements. But Sir James Clark assures us (in his book on consumption) that this is not the case. He has carefully studied the London bills of mortality, making annual averages for periods of ten years, to avoid the influence of epidemics and accidental agencies, and he finds that from 1700 to about 1830, there was no diminution in the frequency and fatality of the disease, but rather that the proportion of deaths from it has been increasing during that whole period. At the same time, this author fully confirms the statement already quoted from the history of England, by showing that the mortality from all diseases, consumption included, has diminished nearly one-half; consumption excluded, more than one-half. I need hardly add, that the profession has never claimed great control over this affection, and that during all the period here referred to, it was held to be incurable. This statement favors a conviction that the advantages we have gained over disease are more in actual practice than in prevention and hygiene.

But we have facts more directly to my purpose, such as will show the physician's care of the sick, freed from all other agencies that are supposed to have influence in prolonging life; and comparing the results of that care, at different periods, our claims will in no respect be weakened.

Dr. Merriman deduces from the bills of mortality just referred to, the fullest evidence, that in the department in which he was so much distinguished, the most signal improvement has been made. In 1680, *one in forty-four* died while under the care of the medical attendant; within fifty years from that time, only *one in seventy* died under the same circumstances; in another term of fifty years, mortality was reduced to *one in eighty-two;* and in forty years more (the period ending with 1820,) it had fallen as low as *one in one hundred and seven.* Here is a condition in which knowledge and skill are left to work their way unhindered and unhelped. Hygiene has little to do with it; the improvements of society even less. It is nature and the doctor, and how has the doctor triumphed? *Fifty-nine per cent.* of such as must have died in the latter half of the seventeenth century, saved in the progress of about one hundred and fifty years. This is doing something to lift from the sex the heavy weight of the primal curse, and we challenge, in return for it, their kind regard.

Let us now bring our inquiry nearer home. In the city of New York is a medical charity, which receives a liberal sum of money every year from the treasury of the State. It cannot fail to interest those of my hearers who have been appointed the almoners of the public bounty, that the New York Hospital, whose reports are annually presented to the Legislature in this hall, is an illustration of the great truth which I have undertaken to expound. This institution reaches back to the beginning of the period for which I claim the most signal improvements in medical practice. I have before me a table, formed from personal examination of the records of this charity, which exhibits the mortality, together with the number of patients treated, annually since its foundation. The first fifty years of its existence end with 1842. I find that if this term be divided into periods of ten years each, the progressive improvement is uninterrupted; so that while the relation of deaths to admissions in the first fifteen years was *one in seven and seven-ninths*, in the last five years it is *one in eleven and one-eighth.* This is a gain of more than 30 per cent., or thirty-one patients saved alive out of every hundred that formerly would have died. Now here is little besides medical treatment. The growth of the city has not materially improved the site of this institution. The same building is now used that was used when it was opened, though others have been added. The wards were no more crowded through the early years than they were in 1842; the comfort of the patient has been equally cared for at both periods; and it is proper to give emphasis to the statement, that in this important result vaccination has had no part. This inestimable discovery was made, it is true, early in this period of fifty years, but it could in no way have affected this hospital, because small pox has never been admitted into it since its foundation. What then have we here but improvement in the practice of medicine and surgery? And it cannot but be noticed that the result here recorded equals, even exceeds, what is claimed in society at large, from all beneficial causes operating together. It is apparent also, since so much has been gained without the aid of vaccination, that great as is the amount of good done by this discovery, it is far from being the only life-saving agency by which the world has been blessed in the past half century.

